# Support of positive association in family-based genetic analysis between *COL27A1* and Tourette syndrome

**DOI:** 10.1038/srep12687

**Published:** 2015-08-03

**Authors:** Shiguo Liu, Xiaoxia Yu, Quanchen Xu, Jiajia Cui, Mingji Yi, Xinhua Zhang, Yinlin Ge, Xu Ma

**Affiliations:** 1 Genetic Laboratory, The Affiliated Hospital of Qingdao University, Qingdao 266003, China; 2 Prenatal diagnosis center, The Affiliated Hospital of Qingdao University, Qingdao, 266003, China; 3 Department of Biochemistry and Molecular Biology, Qingdao University Medical College, Qingdao, China; 4 Department of oral medicine, The Affiliated Hospital of Qingdao University, Qingdao, 266003, China; 5 Department of psychiatry, Medical College, Qingdao University, Qingdao, 266021, China; 6 Child Healthcare Department, The Affiliated Hospital of Qingdao University, Qingdao, 266003, China; 7 Graduate school, Peking Union Medical College, Beijing, China; 8 National Research Institute for Family Planning, Beijing, 100081, China; 9 World Health Organization Collaborating Centre for Research in Human Reproduction, Beijing, China

## Abstract

Recently, a genome-wide association study has indicated associations between single nucleotide polymorphisms in the Collagen Type XXVII Alpha 1 gene (*COL27A1*) and Tourette syndrome in several ethnic populations. To clarify the global relevance of the previously identified SNPs in the development of Tourette syndrome, the associations between polymorphisms in *COL27A1*and Tourette syndrome were assessed in Chinese trios. PCR-directed sequencing was used to evaluate the genetic contributions of three SNPs in *COL27A1*(rs4979356, rs4979357 and rs7868992) using haplotype relative risk (HRR) and transmission disequilibrium tests (TDT) with a total of 260 Tourette syndrome trios. The family-based association was significant between Tourette syndrome and rs4979356 (TDT: χ2 = 4.804, P = 0.033; HRR = 1.75, P = 0.002; HHRR = 1.32, P = 0.027), and transmission disequilibrium was suspected for rs4979357 (TDT: χ2 = 3.969, P = 0.053; HRR = 1.84, P = 0.001; HHRR = 1.29, P = 0.044). No statistically significant allele transfer was found for rs7868992 (TDT: χ2 = 2.177, P = 0.158). Although the TDT results did not remain significant after applying the conservative Bonferroni correction (p = 0.005), the significant positive HRR analysis confirmed the possibility of showing transmission disequilibrium, which provides evidence for an involvement of *COL27A1*in the development of TS. However, these results need to be verified with larger datasets from different populations.

Tourette syndrome (TS) is one of the most commonly diagnosed, heritable, chronic, neuropsychiatric disorder of school-age children occurring at a frequency of 0.5% to 4%^1^. It is defined by multiple motor tics and one or more vocal tics, occurring many times a day for more than 1 year^2^. The disorder is often concurrent with obsessive-compulsive disorder (OCD) and attention deficit hyperactivity disorder (ADHD)^3^. Additional clinical aspects of this disorder cause serious impairment in important areas of functioning including occurrence of anger episodes, anxiety and mood disorders, and learning and sleeping disturbances^4^. Segregation studies in families and twins with TS have provided strong evidence for the existence of a genetic background associated with a multifactorial mode of inheritance^5^,^6^, and numerous studies aiming to explore genetic susceptibility of TS have been published^7^,^8^,^9^,^10^. However, no causative candidate genes have been identified and the genetics of TS is complex and remains unclear.

In recent years, the genome-wide association study (GWAS) approach has led to the identification of many genetic associations for common complex traits^11^. This model-free approach to gene discovery has led to a greater pathophysiological understanding of many disorders and can improve pharmaco-therapeutic strategies^12^. Recently, Scharf and colleagues performed the first GWAS of TS in 1285 cases and 4964 ancestry-matched controls of European ancestry, including two European-derived population isolates, Ashkenazi Jews from North America and Israel and French Canadians from Quebec, Canada. They found the strongest association was with rs7868992, which is within the Collagen Type XXVII Alpha 1 gene (*COL27A1*), (P = 1.85 × 10^−6^) in European-derived samples^13^.

*COL27A1* maps to chromosome 9q32-33, is approximately 156 kb long and has 61 exons^14^, which encode a long triple helical domain, a carboxyl-terminal propeptide (C-propeptide), and a large globular amino-terminal propeptide (N-propeptide). *COL27A1* is strongly expressed in developing cartilage and weakly expressed in many other tissue types such as skin, stomach, gonad and brain^15^. Several studies have unexpectedly identified novel roles of collagens and collagen-like molecules in the developing vertebrate nervous system^16^. However, the function of *COL27A1* in neural development, and specifically in neural circuitry is poorly understood.

To clarify the global relevance of the SNPs that have been associated with TS, the associations need to be confirmed by independent studies in different ethnic groups. The possible associations between SNPs and TS development should also be validated. The objective of this study was to assess the genetic association of three SNPs in *COL27A1* (rs4979356, rs4979357 and rs7868992) with TS in a Chinese Han population.

## Results

The results showed that the allele and genotype frequencies of rs4979356, rs4979357 and rs7868992 in the parents group were not significantly different from those expected according to Hardy-Weinberg equilibrium (for rs4979356, *χ2* = 0.19 and *p* = 0.66; for rs4979357, *χ*^2^ = 0.24 and *p* = 0.62; for rs7868992, *χ*^*2*^ = 1.05 and *p* = 0.30). For all three polymorphisms, *p* > 0.05, suggesting that the population was genetically balanced and that the samples were from the same Mendelian population.

The linkage disequilibrium (LD) analysis revealed that the 3 SNPs seems not in LD (D′1&2 = 0.538, D′1&3 = 0.541, D′2&3 = 0.586; r^2^1&2 = 0.275, r^2^1&3 = 0.222, r^2^2&3 = 0.248), meaning that the 3 SNPs were inherited independently.

The TDT-HRR results showed significant transmission disequilibrium for rs4979356 allele and genotype frequency (TDT = 4.804, df = 1, P = 0.033; HRR = 1.75, *χ*^*2*^ = 9.19, P = 0.002, 95% CI: 1.217-2.523). Transmission disequilibrium for rs4979357 is also suspected (TDT = 3.969, df = 1, P = 0.053; HRR = 1.84, *χ*^*2*^ = 10.55, P = 0.001, 95% CI: 1.271–2.660). No statistical significance of allele transfer was found for rs7868992 (TDT = 2.177, df = 1, P = 0.158; HRR = 1.27, *χ*^*2*^ = 1.77, P = 0.184, 95% CI: 0.894-1.793) (Table 1 and 2).

To increase the efficiency of the test we effectively enlarged the number of cases by HHRR analysis and similar positive results were found for rs4979356 (HHRR = 1.32, *χ*^*2*^ = 4.90, P = 0.027, 95% CI: 1.032–1.697). Significant differences were also found in genotype and allele frequency for rs4979357 (HHRR = 1.29, *χ*^*2*^ = 4.06, P = 0.044, 95% CI: 1.007–1.650). No association was found for rs7868992 (HHRR = 1.22, *χ*^*2*^ = 2.28, P = 0.131, 95% CI: 0.942–1.577) (Table 3). The TDT results showed a marginally significant association between TS and SNP rs4979356 and rs4979357. However, these results failed to reach the conservative Bonferroni-corrected threshold (P correction = 0.005) in the multiple testing.

## Discussion

Recent advances in decreasing the cost of SNP genotyping and rigorous statistical methodology for analyzing large numbers of samples have made GWAS a feasible method for the genetic study of complex disorders^17^. Scharf and colleagues reported the first GWAS of TS^13^. In a primary meta-analysis of GWAS data from European ancestry samples, the top association was found for rs7868992 within *COL27A1* (P = 1.85 × 10^−6^). A secondary analysis including an additional 211 cases and 285 controls also identified rs7868992 to have the highest association (P = 3.6 × 10^7^). Plumb reported that *COL27A1* plays an important structural role in the pericellular extracellular matrix of the growth plate and that homozygotes for an 87 amino acid deletion exhibit severe chondrodysplasia^18^. To date few studies have reported on the correlation between *COL27A1* and TS (in addition to that of Scharf *et al.*^13^), and no strong evidence can support the view that *COL27A1* is a candidate for TS. Nevertheless, recent studies have suggested the importance of collagens in directing neurite extension and connection in the nervous system^19^. *COL27A1* morphant zebrafish show dysmorphic vertebrae lacking hemal and neural spines and impaired notochord development^20^.

In this study, we evaluated the genetic contributions of rs4979356, rs4979357 and rs7868992 polymorphisms in *COL27A1* using a TDT-HRR design with a total of 260 TS trios. Although the traditional case/control study design has many advantages, results from these studies must be interpreted with caution. The most serious concern is that the results can be explained by spurious associations due to mismatches in ethnicity or geographical regions between the control and patient groups, such as population stratification. Moreover, it seems not to be an issue if the call rate of each SNP is 100% and absence of Hardy Weinberg disequilibrium. However, TDT-HRR analysis is a family-based test, and it can be used to find an association due to linkage with an etiological mutation rather than due to spurious associations. By examining the frequency with which a marker allele is transmitted from parents to affected offspring, we can look for an allele to be transmitted more often than by chance. Such family-based approaches clearly complement the case/control design when doing research on association in multiple studies (especially in different populations, ethnic groups and/or different laboratories).

The TDT test showed that rs4979356 had significant transmission disequilibrium, which indicated that rs4979356 was associated with the etiology of TS development. Similar positive results were found in the subsequent HRR and HHRR analysis for rs4979356 suggesting that allele G and genotype G(+) are risk factors for TS. The results also showed that there might be transmission disequilibrium of rs4979357 in TS because the P value of TDT was approximately equal to the test level of significance, 0.05. However, the TDT test results did not remain significant after applying the conservative Bonferroni correction for multiple testing. The reason may be that the TDT and Bonferroni test are conservative themselves to generate significant results. Moreover, the significant positive HRR analysis of rs4975396 confirmed the possibility of showing transmission disequilibrium which provides evidence for an involvement of *COL27A1* in the development of TS. Similarly, the allele C and genotype C(+) of rs4979357 were also risk factors for TS. According to the primary meta-analysis of GWAS data, the highest association was found for rs7868992. It was disappointing that the TDT results for rs7868992 did not revealed statistical significance of allele transfer in any cases. It’s confirmed by the results of linkage disequilibrium test that the 3 SNPs were inherited independently, and thus, differences in genetic ancestry between Chinese and European may account for the insignificant TDT result of rs7868992.

To the best of our knowledge, this is the first report on association between *COL27A1* and the development of TS in a Han Chinese population, in which a new *COL27A1* variant, rs4979356, shows association with TS. Our study lays the groundwork for the eventual identification of further TS candidate variants in larger cohorts. However, this family-based study performed on 260 trios with TS did not demonstrate the influence of rs7868992 on the incidence of TS, as reported by Scharf *et al.* in 2012^13^. All the trios were genotyped by a direct sequencing method which has a relatively high sensitivity and accuracy rate, so the reliability of the data can be effectively guaranteed. A minimum number of samples is required to ensure the testing power of TDT analysis; hence, it would be appropriate to perform further analysis with a larger sample size to quantify the link between rs7868992 polymorphism and TS.

This study and others have only demonstrated associations between polymorphisms in *COL27A1* and TS at the level of statistics and we look forward to results from functional analyses that will, or will not, verify the associations. These findings give hope for possible TS treatment, based on *COL27A1* as a therapeutic target. Clarification of the genetic mechanism of TS will not only help us understand the etiology of TS and comorbid conditions, such as OCD and ADHD, but will also shed light on neural development and growth of the central nervous system.

## Materials and Methods

### Study population

Study subjects between 5 and 18 years of age with a diagnosis of TS, together with their parents, were recruited from the Affiliated Hospital of Qingdao University and Linyi People’s hospital, China. The TS cases comprised 35 female and 225 male outpatients. All probands were diagnosed independently by two experienced psychiatrists according to the DSM-IV criteria and the TS Classification Study Group. They were assessed by means of neurological examination and mental status examination. Subjects were excluded if they presented with unclear diagnosis or incomplete medical record data. The study protocol was approved by the Human Ethics Committee of the Affiliated Hospital of Qingdao University and the National Research Institute for Family Planning and is compliant with the 1975 Declaration of Helsinki. Informed written consent was obtained from every participant or their legal guardians after a complete and extensive explanation of the study. All experiments were performed in accordance with the approved guidelines.

### Laboratory methods

Blood samples were collected from TS patients and their parents, and stored at −20 °C until analyzed. Genomic DNA was extracted from peripheral blood leukocytes according to standard methods. The polymorphic alleles of *COL27A1* were identified by polymerase chain reaction (PCR). The DNA fragment containing the three polymorphisms (rs4979356, rs4979357, rs7868992, which are located close to each other) was amplified using the following primers: 5′-AGACAGGCTGCCTAGTGT-3′ and 5′-GATAGCGTCATTGAACTCC-3′. PCR reactions were carried out in a final volume of 20 μL, containing 2× PCR MasterMix, 0.4 mol/L of each primer and 100 ng of genomic DNA. The reaction was carried out as follows: 94 °C for 5 min, followed by 35 cycles of 95 °C for 30 s, 56 °C for 30 s, 72 °C for 1 min and 72 °C for 10 min. Amplified PCR products were purified and sequenced using the appropriate PCR primers and the BigDye Terminator Cycle Sequencing kit (Applied Biosystems, Foster City, CA, USA) and run on an automated sequencer, a ABI 3730XL (Applied Biosystems), to determine genotype.

### Statistical analysis

All data analyses were carried out using the Statistical Package for the Social Sciences (Windows Version 12.0; SPSS, Chicago, IL, USA). The degree of LD between the adjacent SNPs were estimated using HAPLOVIEW 4.0 (available at http://www.broadinstitute.org/haploview), in which the transmitted and untransmitted group served as case and control group respectively. For trio data from the 260 TS cases, a family-based study was performed to assess genetic association by means of haplotype relative risk (HRR) and transmission disequilibrium tests (TDT) statistics. To increase efficiency of the test, we effectively enlarged the number of cases by haplotype-based haplotype relative risk (HHRR) analysis. In order to deal with the multiple testing problem (TDT–HRR test), we performed a Bonferroni correction test. The conventional P value of 0.05 was divided by the total number of tests performed in the present study (10 tests) accounting for a new P value threshold of 0.005. The 10 tests were calculated as follows: Some studies are done with experiment-wide significance, and here 3 SNPs were examined with three tests each (albeit correlated tests). Thus formally, a Bonferroni correction of 0.05/10 = 0.005 was set for experiment-wide significance, and still a chance observation cannot be totally ruled out.

## Additional Information

**How to cite this article**: Liu, S. *et al.* Support of positive association in family-based genetic analysis between *COL27A1* and Tourette syndrome. *Sci. Rep.* **5**, 12687; doi: 10.1038/srep12687 (2015).

## Figures and Tables

**Table 1:** TDT test results of three genetic loci in 260 trios.

Non-transmitted allele	rs4979356			rs4979357			rs7868992		
		G	C		C	T		A	G
Transmitted allele	G	82	145	C	87	145	A	54	133
	C	110	183	T	113	175	G	110	223
TDT results	χ2 = 4.804, P = 0.033			χ2 = 3.969, P = 0.053			χ2 = 2.177, P = 0.158		

**Table 2:** The HRR results of three genetic loci in 260 trios.

Group	rs4979356(C > G)		rs4979357(T > C)		rs7868992(G > A)	
	G(+)	G(−)	C(+)	C(−)	A(+)	A(−)
Transmitted allele	185	75	190	70	156	104
Non-transmitted allele	152	108	155	105	141	119
Results (χ^2^, P, HRR)	9.19, 0.002, 1.75		10.55, 0.001, 1.84		1.77, 0.184, 1.27	
95%CI	1.217–2.523		1.271–2.660		0.894–1.793	

**Table 3:** The HHRR results of three genetic loci in 260 trios.

Group	rs4979356(C > G)		rs4979357(T > C)		rs7868992(G > A)	
	G	C	A	C	A	G
Transmitted allele	227	293	232	288	187	333
Non-transmitted allele	192	328	200	320	164	356
Results(χ^2^, P, HHRR)	4.90, 0.027, 1.32		4.06, 0.044, 1.29		2.28, 0.131, 1.22	
95% CI	1.032–1.697		1.007–1.650		0.942–1.577	
